# Study on the Correlation between Pain and Cytokine Expression in the Peripheral Blood of Patients with Bone Metastasis of Malignant Cancer Treated Using External Radiation Therapy

**DOI:** 10.1155/2022/1119014

**Published:** 2022-07-08

**Authors:** Yaling Lou, Yu Chen, Yumei Yuan, Ronghua Wang, Hanmin Shan

**Affiliations:** ^1^Department of Clinical Pharmacy, Huzhou Central Hospital, Huzhou, China; ^2^Department of Clinical Laboratory, Huzhou Central Hospital, Huzhou, China; ^3^Department of Pain Treatment, Huzhou Central Hospital, Huzhou, China

## Abstract

The incidence of cancer is increasing worldwide on a yearly basis, with the number of patients with bone metastases also increasing annually. Events associated with bone metastases can seriously affect patient quality of life, through pain, hypercalcemia, bone marrow regeneration disorders, and spinal cord compression. In this nonrandomized controlled clinical trial study, we focused on the relationship between bone metastasis, pain, and cytokines before and after radiotherapy. We hypothesized that radiotherapy alters the cytokine profile of the local bone environment. Combined with the analgesic effects of radiotherapy, certain cytokines may be very sensitive to radiation. External radiation therapy is commonly used to treat cancer patients with bone metastases and can effectively relieve metastasis-related pain, although its underlying mechanisms have not been fully elucidated. For this case-control study, we recruited 30 cancer patients with bone metastasis and 30 healthy individuals. Peripheral venous blood from healthy individuals was collected. The clinical characteristics and peripheral venous blood were collected from patients one week before and one week after radiotherapy. The preradiotherapy and postradiotherapy pain scores, quality of life (QOL), and blood cytokine profiles of the patients to that of the controls were collected to identify pain-related cytokines. Finally, the pain score and the quality of life score improved significantly after radiotherapy. Moreover, the preradiotherapy and postradiotherapy blood cytokine profiles of the patients showed significant differences, indicating that the analgesic effect of radiotherapy against bone metastases is mediated via altered cytokine production. Furthermore, some cytokines were more sensitive to radiotherapy. The levels of MIP-1*δ*, MCP-2, TIMP-1, RANTES, IGFBP3, and TNF-*α* showed significant differences in the pairwise comparative analysis and may therefore mediate pain associated with bone metastasis.

## 1. Introduction

The incidence of cancer is increasing worldwide on a yearly basis, with around 18.1 million new cases diagnosed globally in 2018 [1] and 19.3 million in 2020 [2]. Breast cancer is the most commonly diagnosed cancer with about 2.3 million new cases (11.7%) diagnosed each year, followed by lung cancer (11.4%), colorectal cancer (10.0%), prostate cancer (7.3%), and stomach cancer (5.6%). These cancers are frequently associated with bone metastases. Moreover, the frequency of bone metastasis in the breast, lung, and prostate is the highest among all the solid tumors at 65–70%, and bone metastasis has been associated with pathological fractures, pain, hypercalcemia, bone marrow regeneration disorders, and spinal cord compression [3], all of which significantly affect the quality of life (QOL). Therefore, the treatment of bone metastasis and prevention of bone metastasis-related events in cancer patients is of particular concern.

In this study, various growth, molecules, and chemokines were found to regulate bone metastasis in cancer and are known as cytokines. We investigated changes in the cytokine levels of cancer patients with bone metastasis pain before and after radiotherapy. As far as we know, no similar studies have been published thus far. The research shows that the bone microenvironment not only provides a scaffold for the growth and spread of cancer cells but also secretes a large number of growth factors and cytokines that are critical to bone metastasis [4]. Various cytokines remain in equilibrium in the local microenvironment and are part of a vicious cycle. Radiotherapy is used for the treatment of bone metastases and is not only the most effective method of pain relief but can also induce bone healing and improve the surface of the bone [5]. Hortobagyi followed up on breast cancer patients with bone metastasis for one year and found that 33% of patients needed radiotherapy [6]. Radiation therapy can effectively relieve pain caused by bone metastasis [7], although its mechanisms have not been fully elucidated. However, radiation can be used as a form of physical therapy to induce immunological responses, especially cytokine storms [8]; when the equilibrium in the local microenvironment has been broken, various cytokines show different degrees of fluctuation. We hypothesized that radiotherapy alters the cytokine profile of the local bone environment. Combined with the analgesic effects of radiotherapy, certain cytokines may be very sensitive to radiation. Cytokines play an important role in radiation therapy for cancer patients. Based on this finding, researchers have attempted to verify the relationship between cytokines and clinical characteristics to develop novel cancer treatment regimens and methods of immuno-radiotherapy [9]. In this study, we analyzed the correlation between the preradiotherapy and postradiotherapy levels of different cytokines in peripheral blood of cancer patients with bone metastasis and the extent of postradiotherapy pain relief to identify cytokines involved in metastasis-related pain. Our findings provide novel insights into the role of cytokines in bone metastasis-related pain.

## 2. Methods

### 2.1. Patients

The subjects were divided into the following groups: A, preradiotherapy; B, postradiotherapy; and C, healthy controls. The inclusion criteria for all patients were as follows: (1) no previous chemotherapy and radiotherapy within 4 weeks, and (2) diagnosis of bone metastases based on radio-emission computed tomography or positron emission computed tomography uptake and evaluation of bone destruction using computed tomography or magnetic resonance imaging (MRI). Patients with infectious diseases, immune system diseases, and psychiatric diseases or a history of long-term use of glucocorticoids and nonsteroidal anti-inflammatory drugs were excluded. Due to the strict inclusion criteria and high cost of research, 30 patients who met the inclusion criteria were enrolled. Although 30 is a small sample size, it is the minimum number of samples statistically allowed [10]. None of the subjects from each group were lost. In addition, thirty healthy individuals of age ranging from 18 to 60 years were recruited from the physical examination center and had no history of chronic diseases or long-term medication.

### 2.2. Cytokine Array

Peripheral venous blood was collected from the healthy individuals and from patients one week before and one week after radiotherapy. The samples were collected into tubes containing EDTA and centrifuged at 3000 rpm for 5 min at 4°C. The upper serum layer was aspirated and stored at −80°C. The protein concentration was determined using a BCA Protein Assay Kit (KangChen KC-430, Shanghai Kangcheng Bioengineering Co., Ltd., China).

The Human Cytokine Array G5 membranes (RayBiotech #AAH-CYH-G5, Shanghai Kangcheng Bioengineering Co., Ltd., China) were designed to detect 80 human cytokines. The membranes were incubated in a blocking buffer for 30 min and, thereafter, with the samples at room temperature for 1-2 h or overnight at 4°C. The samples were then decanted, and the membranes were rinsed using a washing buffer and were incubated with diluted biotin-conjugated antibodies at room temperature for 1-2 h. After washing once, the membranes were incubated with streptavidin-conjugated fluor at room temperature, washed again, and scanned using the Axon scanner. The signal intensities were quantified using densitometry to assess the relative expression levels of the cytokines and normalized to median values after eliminating the background signals. Fold changes in protein expression were calculated.

### 2.3. Statistical Analysis

All data were analyzed using SPSS 23.0 statistical software and were found to be normally distributed. Pairwise comparisons were performed using Student's *t*-test, and analysis of variance (one-way ANOVA) was used to test the homogeneity of variance and compare the results between multiple groups. Correlation analysis was performed using Pearson's or Spearman's correlation test. *P* < 0.05 was considered to indicate statistical significance. The *P* values were calculated up to four decimal places; if the *P* value was smaller than 0.0001, the *P* value was reported as *P* < 0.001.

## 3. Results

### 3.1. Clinical Characteristics of Patients

A total of 30 cancer patients with bone metastasis-related pain were recruited from September 2018 to August 2021. All patients were diagnosed based on clinical, radiological, and cytological examinations, and the level of pain was assessed using a numeric rating scale (NRS).

**Figure 1:**
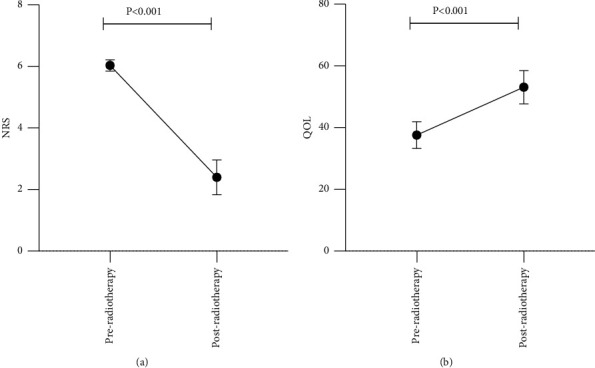
Comparison of pain and quality of life preradiotherapy and postradiotherapy. (a) Analysis of NRS preradiotherapy and postradiotherapy. (b) Analysis of QOL preradiotherapy and postradiotherapy.

The patient cohort was composed of 14 females and 16 males, and the mean age of the patients was 62.9 ± 9.1 years (Table 1). The healthy controls included 10 females and 20 males with a mean age of 40.5 ± 11.1 years, who had no chronic diseases or a long-term medication history. In this study, due to the inclusion of cancer patients, normal levels of cytokine expression were no longer available, and the cytokine expression level of the healthy group was used as the baseline to measure whether the cytokine level of tumor patients increased or decreased before and after radiotherapy. External radiation therapy began once pain could be controlled using drugs. Both the pain score (odds ratio (OR): −3.500, 95% confidence interval (CI): 2.892–4.108, *P* < 0.001) and QOL (OR: −15.330, 95% CI 13.440–17.230, *P* < 0.001) score improved significantly after radiotherapy (Figure 1).

### 3.2. Identification of Pain-Related Cytokines

**Figure 2:**
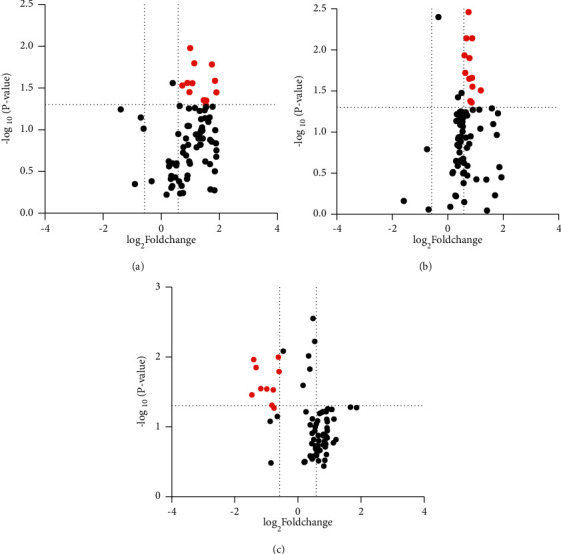
Volcano plots showing levels of the differentially expressed cytokines. (a) Group A vs. group C. (b) Group B vs. group C. (c) Group B vs. group A. The red dots indicate a fold change of ≥1.5 or ≤−1.5 and a *P* value of ≤0.05, while the black dots indicate that the result was not significant.

**Figure 3:**
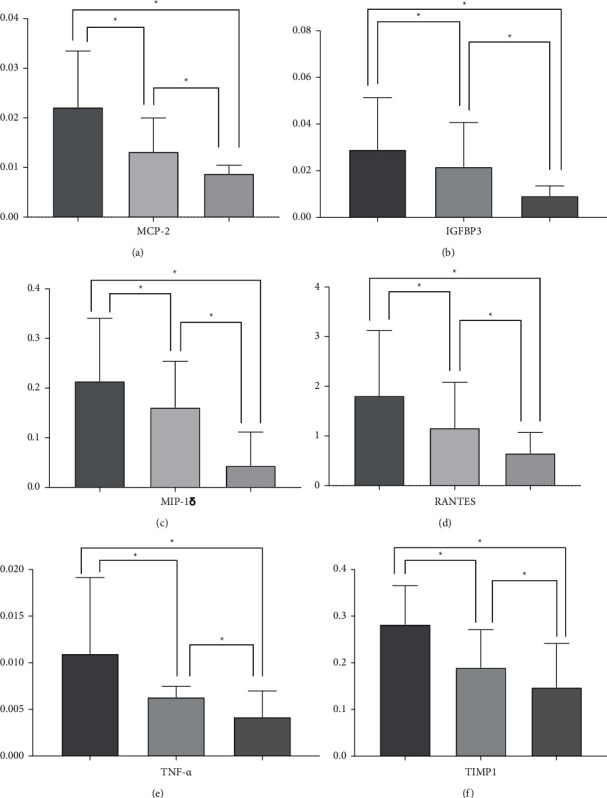
Pairwise comparisons were performed using Student's *t*-test. Comparisons of MIP-1*δ*, MCP-2, TIMP-1, RANTES, IGFBP3, and TNF-*α* levels between the three groups. The *y*-axis represents a standardized cytokine expression level and ^*∗*^represents a significant difference. MCP—*P*=0.0021, preradiotherapy vs. postradiotherapy; *P*=0.0155, postradiotherapy vs. healthy controls; *P* < 0.001, preradiotherapy vs. healthy controls. IGFBP3—*P*=0.0067, preradiotherapy vs. postradiotherapy; *P*=0.0097, postradiotherapy vs. healthy controls; *P* < 0.001, preradiotherapy vs. healthy controls. MIP-1*δ*—*P*=0.0067, preradiotherapy vs. postradiotherapy; *P*=0.0097, postradiotherapy vs. healthy controls; *P* < 0.001, preradiotherapy vs. healthy controls. RANTES—*P*=0.0019, preradiotherapy vs. postradiotherapy; *P*=0.0417, postradiotherapy vs. healthy controls; *P*=0.0263, preradiotherapy vs. healthy controls. TNF-*α*—*P*=0.0019, preradiotherapy vs. postradiotherapy; *P*=0.0417, postradiotherapy vs. healthy controls; *P*=0.0263, preradiotherapy vs. healthy controls. TIMP-1—*P*=0.0054, preradiotherapy vs. postradiotherapy; *P*=0.0466, postradiotherapy vs. healthy controls; *P*=0.0234, preradiotherapy vs. healthy controls.

The expression levels of pain-related cytokines were detected using the Human Cytokine Array G5 antibody chip, which can be used to detect 80 different cytokines (Table 2). The differentially expressed cytokines between the distinct pairs were identified through volcano plots with a fold change of ≥1.5 or ≤ −1.5 and a *P* value of ≤0.05 as the thresholds. As shown in Figure 2, 12 differentially expressed cytokines were found between group A and group C, 12 between group B and group C, and 10 between group B and group A (Table 3, Figure 2). Fifteen of the 34 differentially expressed cytokines were common to all three pairs. Of the remaining 19 cytokines, MIP-1*δ*, MCP-2, TIMP-1, RANTES, IGFBP3, and TNF-*α* were expressed in all groups and showed significant differences (Figure 3). The differentially expressed cytokines in patients with primary lung, colorectal, liver, ovarian, bladder, renal, breast and prostate tumors are given in Table 4.

Pairwise comparison was performed using Student's *t*-test. *A* represents the log_2_ (fold change) of ratio of preradiotherapy and healthy controls, *B* represents the log_2_ (fold change) ratio of postradiotherapy and healthy controls, and *C* represents the log_2_ (fold change) ratio of preradiotherapy and postradiotherapy.

If the confidence level was 95%, the true percentage of the population is 50%, the total sample size is 30, and therefore, the minimum sample size of primary cancer is 15. If the sample number is less than 15, the level of credibility is too low and the *P* value is meaningless. The relationship between changes in cytokine levels before and after radiotherapy and the primary tumor was not statistically significant. Fortunately, the number of lung cancer patients was greater than 15, and changes in cytokine levels before and after radiotherapy were statistically significant, although the true percentage of the population was only 50%.

## 4. Discussion

Bone metastases are extremely common in cancer, and pain is a frequent complication that significantly affects the quality of life and survival of patients. The aim of our study was to screen for cytokines involved in bone metastasis-related pain in cancer patients to identify novel targets to manage pain and overcome resistance against local treatment. The bone metastasis of cancer cells has a complex and heterogeneous pathogenesis, and the most common manifestation is osteolysis and osteogenesis. The circulating tumor cells are adsorbed to the bone surface, where they secrete several cytokines, including transforming growth factor (TGF)-*β*, which allows the cells to survive and proliferate into the bone microenvironment [11]. In addition, growth factors and cytokines are also released by osteoblasts and bone tissue following osteoclast-mediated destruction, including bone-derived growth factors, free calcium, CXCR4, IL-11, TNF-*α*, matrix metalloproteinase, parathyroid hormone-related protein, RANKL, and insulin-like growth factor 1, all of which stimulate tumor growth and metastasis. The tumor-derived cytokines in turn stimulate osteolysis, resulting in a vicious cycle of tumor cell proliferation and osteoclast-mediated bone resorption [5].

We found that the degree of pain and quality of life of patients improved significantly after external radiotherapy, which is consistent with the results of other studies [12–14]. Immune cells, such as lymphocytes and macrophages, play a dual role in bone metastasis by secreting specific cytokines [15]. The levels of TNF-*α*, MCP-2, IL-8, and 12 other cytokines were significantly different between the preradiotherapy and healthy groups (*P* < 0.05), while that of TNF-*α*, MCP-2, IL-15, and another 12 cytokines were significantly different between the postradiotherapy and healthy groups (*P* < 0.05). In addition, MIP-1*δ*, MCP-2, TIMP-1, RANTES, IGFBP3, and TNF-*α* expression levels were significantly different between the preradiotherapy and postradiotherapy groups and may thus be closely associated with bone metastasis-related pain. We analyzed the primary tumors and found that the results were inconsistent with the results obtained through the unstratified analysis (Table 4), while the number and type of cytokines also showed large differences. Several types of tumors had only a small number of samples, ranging from 1 to 4 cases. Therefore, no significant results were obtained with regard to cytokines obtained from the stratified analysis in this study. However, MDC, IGFBP4, IGFBP3, MIF, RANTES, TARC, BDNF, NAP-2, TIMP-1, MCP-2, TGF-*β*1, and TNF-*α* were significantly different between the lung cancer preradiotherapy and postradiotherapy groups. MCP-2, TIMP-1, RANTES, IGFBP3, and TNF-*α* also appeared in the results of the unstratified analysis.

MIP-1*δ*, MCP-2, and RANTES belong to the CC family of chemokines that regulate bone remodeling, inhibit antitumor immune responses, promote tumor growth, and induce the proliferation and differentiation of bone marrow-derived inhibitory cells [15, 16]. In addition, RANTES and MCP-2 recruit monocytes and neutrophils to tumors to maintain a chronic inflammatory microenvironment [17]. The chemokine ligand, RANTES, and IL-6 released by tumor cells promote their growth through autocrine and paracrine mechanisms, and the simultaneous expression of both results in a more aggressive phenotype [18]. Cytokines stored in the bone matrix are released during bone destruction and are key to bone-tumor interactions. One study reported a significant increase in IGFBP3 levels in patients with tumor bone metastasis [19]. IGFBP3 regulates the bioavailability of IGF1, which is abundant in the bone microenvironment and regulates osteoclast differentiation. Although IGFBP3 increases the activity of bone marrow macrophages, it inhibits their differentiation into osteoclasts [20]. There is evidence that low MCP-2 and TNF-*α* levels are correlated with a better prognosis and longer progression-free survival of breast cancer patients with bone metastasis [21]. Metalloproteinase tissue inhibitor 1 (TIMP-1) is an inflammatory factor that plays a multipotent role in the bone marrow microenvironment and regulates the survival and proliferation of different cell types, including tumor cells [22]. Some studies have reported elevated levels of TIMP-1 in plasma of leukemia patients [23], which is associated with the proliferation and migration abilities of leukemia cells.

Pain is a common complication of bone metastasis, which significantly affects the quality of life and survival of cancer patients. Although there are many local treatment options that can be used to manage pain, patients may eventually become resistant to these methods. The identification of cytokines associated with pain offers a novel therapeutic strategy for patients with bone metastases since the blocking of the expression of these cytokines can improve pain and the quality of life.

One limitation of our study is the small size of the cohort due to strict inclusion criteria and economic constraints. Therefore, our findings will need to be validated using a larger cohort.

## Figures and Tables

**Table 1:** Clinical characteristics of patients.

No.	Age	Gender	Primary tumor	Antineoplastic agents	Analgesic drugs	Painful bone metastasis	Radiotherapy protocol
1	67	Female	Lung cancer	Icotinib	Tramadol, gabapentin, zoledronate	L3/4	10 MV-X line 3D-CRT DT 30 Gy/10 F
2	54	Male	Lung cancer	Osimertinib	Tramadol	C3/4	MLC 3DCR-T-SAD DT 30 Gy/10 F
3	68	Female	Lung cancer	Icotinib	Tramadol	Left ilium, left lip	10 MV-X line 3D-CRT DT 30 Gy/10 F
4	65	Male	renal cancer	Sorafenib	Tramadol	Left ilium	10 MV-X line 3D-CRT DT 30 Gy/10 F
5	69	Female	Lung cancer	Osimertinib	Tramadol	Right whirlbone	MLC 3DCR-T-SAD DT 30 Gy/10 F
6	66	Male	Lung cancer	Bicalutamide	Tramadol, zoledronate	Right lip	MLC 3DCR-T-SAD DT 30 Gy/10 F
7	70	Male	Lung cancer	Icotinib	Zoledronate	Right ilium	10 MV-X line 3D-CRT DT 45 Gy/15 F
8	62	Female	Lung cancer	—	Oxycodone	C4	10 MV-X line 3D-CRT DT 30 Gy/10 F
9	66	Female	Colorectal cancer	Bevacizumab	Tramadol, zoledronate	Left lip	10 MV-X line 3D-CRT DT 30 Gy/10 F
10	65	Female	Gallbladder carcinoma	—	Oxycodone	L3-5	MLC 3DCR-T-SAD DT 30 Gy/10 F
11	56	Female	Liver cancer	Regorafenib	Tramadol, gabapentin, zoledronate	Right ilium	10 MV-X line 3D-CRT DT 30 Gy/10 F
12	67	Male	Renal cancer	—	Tramadol	Right ilium	10 MV-X line 3D-CRT DT 30 Gy/10 F
13	64	Male	Colorectal cancer	—	Tramadol, gabapentin	Right ilium	10 MV-X line 3D-CRT DT 30 Gy/10 F
14	67	Male	Liver cancer	Gefitinib	Tramadol, gabapentin	T11	10 MV-X line 3D-CRT DT 30 Gy/10 F
15	46	Female	Liver cancer	Lenvatinib	Tramadol	S1, left ilium, left whirlbone	10 MV-X line IMRT PTV DT 50 Gy/20 F
16	74	Female	Lung cancer	Gefitinib	Tramadol, zoledronate	L5-S1	10 MV-X line IMRT GTV DT 30 Gy/10 F
17	53	Female	Lung cancer	Icotinib	Tramadol, zoledronate	Right arm	10 MV-X line 3D-CRT DT 30 Gy/10 F
18	38	Female	Ovarian cancer	Bevacizumab	Tramadol	L3	10 MV-X line 3D-CRT DT 30 Gy/10 F
19	57	Male	Lung cancer	Bevacizumab	Tramadol	T12, L1-4	10 MV-X line 3D-CRT DT 30 Gy/10 F
20	74	Female	Lung cancer	Teriprizumab	Oxycodone	L5	10 MV-X line IMRT PTV DT 30 Gy/10 F
21	86	Male	Lung cancer	—	Tramadol	T8/9	10 MV-X line IMRT PTV DT 45 Gy/15 F
22	55	Male	Liver cancer	Sorafenib	Oxycodone, gabapentin	Right clavicle, scapula, humerus	10 MV-X line 3D-CRT DT 30 Gy/10 F
23	61	Male	Prostate cancer	Bicalutamide	Tramadol	T10-L1	10 MV-X line 3D-CRT DT 30 Gy/10 F
24	59	Female	Lung cancer	Osimertinib	Tramadol	Left ilium	10 MV-X line IMRT PTV DT 33 Gy/11 F
25	55	Female	Breast cancer	Anastrozole	Tramadol, zoledronate	T11-L2	10 MV-X line IMRT PTV DT 30 Gy/10 F
26	72	Male	Lung cancer	—	Tramadol	Left femur	10 MV-X line IMRT PTV DT 45 Gy/15 F
27	61	Female	Colorectal cancer	—	Gabapentin	T12, L1-5	10 MV-X line 3D-CRT DT 30 Gy/10 F
28	69	Male	Lung cancer	Bevacizumab	Tramadol	T11/12	MLC 3DCR-T-SAD DT 30 Gy/10 F
29	60	Female	Lung cancer	Gefitinib	Tramadol, zoledronate	C5/6	10 MV-X line IMRT PTV DT 45 Gy/15 F
30	63	Male	Lung cancer	Cindillizumab	Tramadol	S1	10 MV-X line IMRT PTV DT 30 Gy/10 F

**Table 2:** Cytokine array G5 cytokine profile.

No.	Cytokines
1	GDNF
2	IGFBP4
3	ANG
4	PARC
5	MIP-1*β*
6	VEGF-A
7	MCP-4
8	IL-1*β*
9	IGFBP2
10	FGF9
11	IL-15
12	RANTES
13	MCP-1
14	MIP-3*α*
15	CCL26
16	TGF-*β*1
17	OPG
18	TIMP-1
19	IGFBP3
20	PDGF-BB
21	GCP-2
22	SDF-1
23	OPN
24	IGF-I
25	SCF
26	MCP-2
27	THPO
28	EGF
29	IL-12
30	PLGF
31	FGF7
32	MIF
33	IL-3
34	Flt-3 LG
35	IFN-*γ*
36	IGFPBP1
37	TGF-*β*2
38	I-309
39	TIMP-2
40	NT-3
41	IP-10
42	HGF
43	LIGHT
44	FGF6
45	CX3CL1
46	GRO-*α*
47	IL-13
48	NAP-2
49	NT-4
50	BDNF
51	IL-4
52	BLC
53	IL-8
54	CK*β* 8-1
55	FGF4
56	CCL24
57	IL-10
58	1L-16
59	CSF3
60	LEPTIN
61	LIF
62	MIG
63	MDC
64	CCL11
65	IL-1*α*
66	TGF-*β*3
67	IL-2
68	CSF1
69	GRO
70	CSF2
71	MCP-3
72	IL-7
73	MIP-1*δ*
74	IL-5
75	OSM
76	TARC
77	IL-6
78	TNF-*α*
79	ENA-78
80	TGF-*β*

**Table 3:** Comparative analysis between groups of cytokines.

Cytokines	*A*	*B*	*C*	*P*	OR	95% CI
MIP-1*δ*	0.903	—	—	0.0180	0.2143	0.1385–0.2900
MCP-2	1.552	—	—	0.0298	0.0715	0.0087–0.1354
IGFBP3	0.997	—	—	0.0011	0.0064	0.0033–0.0095
FGF9	1.851	—	—	0.0428	0.0119	0.0005–0.0234
RANTES	1.744	—	—	0.0178	0.8100	0.1765–1.4440
TGF-*β*1	1.898	—	—	0.0038	0.0075	0.0031–0.0118
IL-7	1.071	—	—	0.0078	0.0021	0.0007–0.0035
TIMP-1	0.972	—	—	0.0173	0.1000	0.0223–0.1777
BLC	1.465	—	—	0.0435	0.0073	0.0003–0.0143
IL-8	1.537	—	—	0.0494	0.0045	0.0014–0.0088
MDC	0.724	—	—	0.0031	0.0076	0.0033–0.0119
TNF-*α*	1.139	—	—	0.0185	0.0075	0.0014–0.0122
THPO	—	0.863	—	0.0025	0.0031	0.0012–0.0048
TIMP-1	—	0.632	—	0.0159	0.0872	0.0183–0.1560
FGF6	—	0.786	—	0.0087	0.0357	0.0102–0.0612
CSF2	—	1.198	—	0.0490	0.0014	0.0007–0.0027
RANTES	—	0.751	—	0.0408	0.8570	0.0400–1.674
TNF-*α*	—	0.817	—	0.0397	0.0021	0.0001–0.0042
MCP-4	—	0.890	—	0.0460	0.0090	0.0002–0.0178
MIP-1*δ*	—	0.675	—	0.0029	0.1631	0.0916–0.2347
MCP-2	—	0.776	—	0.0455	0.0022	0.0005–0.0043
IGFBP3	—	0.605	—	0.0458	0.0155	0.0003–0.0306
FGF9	—	0.878	—	0.0183	0.0137	0.0026–0.0248
IL-15	—	0.880	—	0.0384	0.0009	0.0005–0.0017
TIMP-1	—	—	−0.779	0.0215	0.0925	0.0153–0.1696
MIP-1*δ*	—	—	−0.619	0.0406	0.0775	0.0037–0.1512
MCP-2	—	—	−1.400	0.0017	0.0134	0.0058–0.0210
IGFBP3	—	—	−0.595	0.0129	0.0198	0.0047–0.0349
RANTES	—	—	−0.986	0.0375	0.4469	0.0287–0.8652
THPO	—	—	−0.818	0.0429	0.0015	0.0005–0.0030
IL-7	—	—	−1.360	0.0287	0.0012	0.0001–0.0023
TARC	—	—	0.755	0.0137	0.0217	0.0050–0.0383
IL-6	—	—	−1.178	0.0075	0.0013	0.0004–0.0022
TNF-*α*	—	—	−1.457	0.0409	0.0056	0.0003–0.0110

**Table 4:** Relationship between cytokines and primary cancer preradiotherapy and postradiotherapy.

	Primary cancer	Cytokines	*P*	OR	95% CI
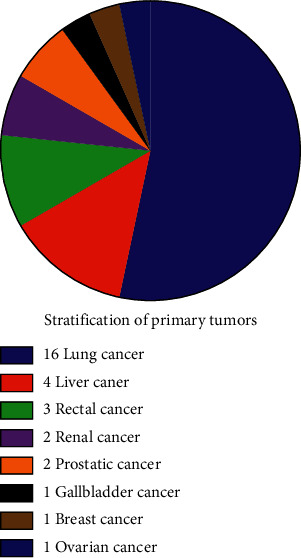	Lung	MDC	0.0276	0.0116	0.0010–0.0149
	Lung	IGFBP4	0.0031	0.0677	0.0187–0.0729
	Lung	IGFBP3	0.0437	0.0245	0.0005–0.0295
	Lung	MIF	0.0424	0.0231	0.0005–0.0249
	Lung	RANTES	0.0066	0.0109	0.0025–0.0127
	Lung	TARC	0.0282	0.0112	0.0009–0.0141
	Lung	BDNF	0.0228	0.1170	0.0110–0.1242
	Lung	NAP-2	0.0103	0.1365	0.0310–0.1909
	Lung	TIMP-1	0.0495	0.0153	0.0002–0.0180
	Lung	MCP-2	0.0158	0.0099	0.0016–0.0126
	Lung	TGF-*β*1	0.0134	0.0056	0.0009–0.0065
	Lung	TNF-*α*	0.0170	0.0124	0.0025–0.0215

## Data Availability

The data used to support the findings of this study are available from the corresponding author upon request.
